# Contamination of Ethiopian paper currency notes from various food handlers with *E. coli*

**DOI:** 10.1186/s40064-016-2742-z

**Published:** 2016-07-12

**Authors:** Adem Hiko, Kasahun Abdata, Yimer Muktar, Mezene Woyesa, Abdela Mohammed

**Affiliations:** College of Veterinary Medicine, Haramaya University, Po. Box. 138, Dire Dawa, Ethiopia; School of Veterinary Medicine, Collage of Medical and Health Sciences, Wollega University, Po. Box 395, Nekemte, Ethiopia

**Keywords:** Antimicrobial, *E. coli* contamination, Food handlers, Public health, Haramaya, Paper currency

## Abstract

Contamination rate of Ethiopian paper currency notes handled by various food handlers with *Escherichia coli* and antimicrobial susceptibility of the isolates was assessed. A total of 384 Ethiopian Birr (ETB) notes were randomly sampled from meat handlers at butchers, bread and the related food handlers at cafeteria, fruit and vegetables handlers at supermarket, and milk sellers both at open market and dairy station. Fifty control new currencies were also sampled from Commercial Bank of Ethiopia. Both surfaces of the currency were swabbed using wet sterile cotton. The swab was overnight incubated in buffered peptone water. A loop full was streaked on eosin methylene blue agar and followed by biochemical test on presumptive *E. coli* colonies. Randomly selected isolates were exposed to chloramphenicol (C-30 µg), neomycin (N-30 µg), oxytetracycline (OT-30 µg), polymyxin-B (PB-300 IU) and trimethoprim-sulfamethoxazole (SXT-1.25/23.75/µg) susceptibility using disc diffusion techniques. *E. coli* was not isolated from currency used as control. A total of 288 (75 %) currency notes were found carrying *E. coli*. *E. coli* prevalence was ranges from 67.2 % at open market milk sellers to 87.2 % at dairy station milk sellers; from 64.8 % on ETB 100 to 82.9 % on ETB 1. Differences were not observed in *E. coli* prevalence on currency notes from among almost all food handlers (P > 0.05). Susceptibility of tested isolates to each chloramphenicol, oxytetracycline and trimethoprim-sulfamethoxazole was 100 %, and to polymyxin-B was 97.3 %. High resistance (83.7 %) was observed to neomycin. The finding indicates, contaminated food can be a source of *E. coli* for further contamination of currency which again transfer through various foods ready for consumption.

## Background

Currency notes are used for exchange of commodity and goods, including food. While so, it could be contaminated and represents as a vehicle for the transmission of pathogenic microorganisms in the environment and among humans (Michaels 2002; Xu et al. 2005). The Ethiopian currency called “Birr” is the second most used currency in Africa after Nigerian “Naira” for goods and services, including food and others, exchange in Ethiopia as in most other countries worldwide (Girma et al. 2014; Saripalli et al. 2014). The currency notes can be contaminated while counting using saliva, coughing and sneezing on hands followed by exchanging currency, placement or storage on dirty surfaces, poor hand washing post toilet. Such currency notes further act as a vehicle for bacteria to the next user (Barolia et al. 2011; Ngwai et al. 2011). Simultaneous handling of food and such contaminated currency could resulted in foodborne infection (Girma et al. 2014; Ngwai et al. 2011). Microbial contamination rate of currency handled by butchers (78.0 %) and food sellers (62.1 %) was also reported from Nepal (Lamichane et al. 2009). Microbial load of *Bacilli*, *Coccus*, *Fungal* species (Saripalli et al. 2014) from Axum-Ethiopia, *Staphylococcus,Enterobacteriaceae,Micrococcus* species, *Streptococcus* species (Girma et al. 2014) from Jimma-Ethiopia were reported from Ethiopian Birr. The Food and Agricultural Organization (2011) listed the possible means of food contamination and/or cross-contamination during growth and harvest (horticulture products), collection (milk) or slaughter (meat). Further contamination can occur during post-harvest handling, transport, processing and unhygienic food handling during preparation (FAO 2011). *Escherichia coli* (*E. coli*) was reported from currency at 5.6 % from Ghanaian Currency Notes (Tagoe et al. 2009). However, *E. coli* carriage of Ethiopian currency notes handled by various types of food handlers was not yet assessed and the isolates were not also tested for drug susceptibility in and around Haramaya district. Thus, the aim of this study was to assess contamination rate of Ethiopian polymer currency notes handled by various types of food handlers with *E. coli* in and around Haramaya district with antimicrobial susceptibility test on the selected isolates.

## Methods

### Sampling

A study was conducted at Haramaya district in east Hararghe Zone of Oromia Regional State, Ethiopia to determine the prevalence of *E. coli* on Ethiopia Birr (ETB) paper currency notes handled by various food type handlers with *E. coli*. Simple random sampling technique was applied both on the food handlers and the notes types of currency at Haramaya University (HU) and the surrounding, and at Haramaya town.

### Sample sources

The sample of currency was collected from meat seller at butcher, milk handler both at open market and dairy station, fruit and vegetables sellers’ at supermarket, and bread and the related seller’s at cafeteria. A random of Birr 1, Birr 5, Birr 10, Birr 50, and Birr 100 currency notes were collected from each notes. Age and sex of handlers were also considered. Using 95 % confidence interval (CI) with required 5 % precision (Thrusfield 2005) sample size was calculated. A total of 384 ETB notes were sampled. During each sampling occasion, 10 each ETB currency notes were directly collected from Automatic Teller Machines (ATM) and Commercial Bank of Ethiopia branch in the study area for control purpose.

### Sample collection

All samples were collected aseptically by letting the selected individuals to drop a selected currency note into sterile polythene bags while substitution with equal currency. The polythene bags were promptly sealed, immediately labeled and transported to Microbiology Laboratory, College of Veterinary Medicine, Haramaya University (HU) for microbiological analysis.

### Laboratory procedures

Both surfaces of each sampled currency were thoroughly swabbed aseptically using sterile Buffered Peptone Water (BPW) (Merck, Germany) soaked cotton on pre-sterilized aluminum foil. The swab was dipped into 10 ml BPW and incubated for 24 h at 37 °C. After which a loop full was streaked on Eosin Methylene Blue (EMB) agar (Oxoid, UK) and incubated overnight at 37 °C. Characteristic metallic shine *E. coli* presumptive colony was transferred into Nutrient Agar for biochemical test using Indole production, Methylene red reduction, Voges Proskauer reaction and Citrate utilization (IMViC) tests according to Quinn et al. (2004). *Escherichia coli* ATCC^®^ 25922™ (USA) was used as a control cultures. Antimicrobial susceptibility test was done on 43 randomly selected isolates by the Kirby-Bauer disk diffusion method (Bauer et al. 1966; CLSI 2007). The tested bacterium was taken from an overnight freshly grown culture and inoculated into 5 ml Brain Heart Infusion Broth (Merck, Germany). The inoculated broth was incubated for 4 h to approximately 10^6^ CFU/ml at McFarland 0.5 % level of turbidity (CLSI 2007). With this culture, a bacterial lawn was prepared on Mueller–Hinton agar (Oxoid, UK). Antimicrobials against (Oxoid, UK) including chloramphenicol (C 30 µg), neomycin (N 30 µg), oxytetracycline (OT 30 µg), polymyxin-B (PB 300 IU) and trimethoprim-sulfamethoxazole (SXT 1.25/23.75/µg) were used for test. Result was interpreted using the diameter of zone of bacterial growth inhibition surrounding the disc (CLSI 2007).

### Data analysis

The data obtained from laboratory examination was recorded in Microsoft excel 2007, analyzed using STATA 11 version and IBM SPSS 20. Percentage was calculated to evaluate for *E. coli* prevalence at all study variables. Significances of associated study factors/variables were compared using one way ANOVA. Significance of differences was considered at 95 % confidence interval (P < 0.05) for study variables such as sample sources, currency note denomination and food types for *E. coli* positive.

## Results

An overall 75.0 % of ETB were found to contain *E. coli*. *E. coli* carriage rate of 73.8 and 77.1 % were observed on currency from Haramaya University and the surrounding, and Haramaya town respectively. The contamination ranges from 67.2 % at open market to 87.2 % at dairy station milk sellers. With regards to currency notes, presence of *E. coli* ranged from 64.8 % on the ETB 100 notes to 82.9 % on ETB 1 notes. The prevalence of *E. coli* showed no any difference among and between all variables of study including sex and age categories food handlers (Table 1). However, no *E. coli* contamination was observed in control currency.

**Table 1:** Rate of ETB contamination with *E. coli* among studied risk variables of food handlers

Variables	No. of examined	Positive no. (%)	*P* value
Location			
HU and surrounding	240	177 (73.8)	*P* > 0.05
Haramaya town	144	111 (77.1)	
Source–food type handled			
Butcher—meat handlers	92	68 (73.9)	*P* > 0.05
Open market— milk sellers	58	39 (67.2)	
Dairy station—milk sellers	39	34 (87.2)	
Cafeteria—Bread and relate handlers	92	70 (76.1)	
Supermarket—Fruit and vegetables handlers	103	77 (74.8)	
Currency notes			
Birr 1	82	68 (82.9)	*P* > 0.05
Birr 5	81	61 (75.3)	
Birr 10	77	60 (77.9)	
Birr 50	73	53 (72.6)	
Birr 100	71	46 (64.8)	
Handlers age in years			
11–20	123	95 (77.2)	*P* > 0.05
21–30	167	127 (76.1)	
31–40	94	66 (70.2)	
Handlers sex			
Female	192	143 (74.5)	*P* > 0.05
Male	192	145 (75.5)	
Total	384	288 (75.0)	

In particular, at Haramaya University and the surrounding, currency contamination was 73.8 % with low (47.1 %) at open market and higher (87.2 %) at dairy station milk sellers showing significant difference (P < 0.05). However, among currency notes, age and sex, contamination ranged from 60.9 % on ETB 100 to 85.4 % on ETB 1 with no any differences (P > 0.05) within variables of study (Table 2).

**Table 2:** *E. coli* on ETB among studied risk variables of food handlers at Haramaya University and the surrounding

Variables	No. of examined	Positive no. (%)	*P* value
Source—food type handled			
Butcher—meat handlers	62	44 (71.0)	*P* < 0.05
Open market—milk sellers	17	8 (47.1)	
Dairy station—milk sellers	39	34 (87.2)	
Cafeteria—Bread and relate handlers	59	43 (72.9)	
Supermarket—Fruit and vegetables handlers	63	48 (76.2)	
Currency notes			
Birr 1	48	41 (85.4)	*P* > 0.05
Birr 5	53	40 (75.5)	
Birr 10	43	32 (75.5)	
Birr 50	50	36 (72.0)	
Birr 100	46	28 (60.9)	
Handlers age in years			
11–20	81	67 (82.7)	*P* > 0.05
21–30	94	67 (71.3)	
31–40	65	43 (66.2)	
Handlers sex			
Female	114	84 (73.7)	*P* > 0.05
Male	126	93 (73.8)	
Total	240	177 (73.8)	

With regards to Haramaya town, *E. coli* prevalence ranges from 72.5 % on currency obtained from fruit and vegetables handlers at supermarket to 81.8 % on that of from bread and related food handlers at cafeteria. Here, the prevalence was ranges from 72.1 % on ETB 100 to 82.4 % on ETB 10 among currencies notes. Difference among and between factors/variables within all variables of study were not observed (P > 0.05) (Table 3).

**Table 3:** *E. coli* on ETB among studied risk variables of food handlers at Haramaya town

Variables^a^	No. of examined	Positive no. (%)	*P* value
Source–food type handled			
Butcher—meat handlers	30	24 (80.0)	*P* > 0.05
Open market—milk sellers	41	31 (75.6)	
Cafeteria—Bread and relate handlers	33	27 (81.8)	
Supermarket—Fruit and vegetables handlers	40	29 (72.5)	
Currency notes			
Birr 1	34	27 (79.4)	*P* > 0.05
Birr 5	28	21 (75.0)	
Birr 10	34	28 (82.4)	
Birr 50	23	17 (73.9)	
Birr 100	25	18 (72.1)	
Handlers age in years			
11–20	42	28 (66.7)	*P* > 0.05
21–30	73	60 (82.2)	
31–40	29	23 (79.3)	
Handlers sex			
Female	78	59 (75.6)	*P* > 0.05
Male	66	52 (78.8)	
Total	144	111 (77.1)	

^a^Dairy station was not available

Susceptibility of selected and tested *E. coli* isolates from ETB notes to antimicrobial agents of under investigation was showed in the Table 4 below. It indicates that isolates from all variables was 100 % susceptible to each of chloramphenicol, oxytetracycline and trimethoprim-sulfamethazole while there was frequent resistance to neomycin (83.7 %) without double and multiple drug resistance strains.

**Table 4:** Antimicrobial susceptibility/profiles of contaminant *E. coli* from ETB

Variables	No. of *E. coli* tested	C No. (%)^a^	OT No. (%)^a^	SXT No. (%)^a^	PB No. (%)^b^		N No. (%)^c^		
		S	S	S	S	R	S	I	R
Food handlers and source									
Meat handlers at butcher	8	8 (100)	8 (100)	8 (100)	7 (87.5)	1 (12.5)	–	2 (25)	6 (75)
Milk seller at open market	9	9 (100)	9 (100)	9 (100)	9 (100)	–	2 (22.2)	–	7 (77.8)
Milk seller at dairy station	2	2 (100)	2 (100)	2 (100)	2 (100)	–	–	–	2 (100)
Bread and the related food handler at cafeteria	10	10 (100)	10 (100)	10 (100)	10 (100)	–	–	–	10 (100)
Fruit and vegetables handlers at supermarket	14	14 (100)	14 (100)	14 (100)	14 (100)	–	–	3 (21.4)	11 (78.6)
Currency									
Birr 1	9	9 (100)	9 (100)	9 (100)	9 (100)	–	–	–	9 (100)
Birr 5	10	10 (100)	10 (100)	10 (100)	9 (90)	1 (10.0)	1 (10.0)	2 (20.0)	7 (70.0)
Birr 10	9	9 (100)	9 (100)	9 (100)	9 (100)	–	1 (11.1)	–	8 (88.9)
Birr 50	8	8 (100)	8 (100)	8 (100)	8 (100)	–	–	3 (37.5)	5 (62.5)
Birr 100	7	7 (100)	7 (100)	7 (100)	7 (100)	–	–	–	7 (100)
Total	43	43 (100)	43 (100)	43 (100)	42 (97.7)	1 (2.3)	2 (4.7)	5 (11.6)	36 (83.7)

^a^All are susceptible, ^b^ Intermediate was not observed, ^c^ Susceptible, intermediate and resistance was observed

*S* susceptible, *I* intermediate, *R* resistance

## Discussion

The present of *E. coli* on ETB at high level (75 %), and handled by five food classes (meat handler, open market milk seller, dairy station milk seller, bread and related food handlers, and fruit and vegetable sellers) at different locations indicates the likelihood of public infection with harmful microbial including pathogenic *E. coli* strain in the studied area. It was higher than the 50 % reported from Nigeria by Ngwai et al. (2011) in currency from samples including non-food items. This could be due to either contamination of the currency from food that had already contaminated with feces or others sources of *E. coli*. The moisture, sweat, air and others factors from the food at ambient temperature are driving force for contamination and survival of *E. coli* on the currency with risk of further contamination of food at any point. Lamichane et al. (2009) and Saripalli et al. (2014) also provided similar suggestion. Jay et al. (2005) also indicated the possible risk of food cross contamination at any point. Moreover, the present finding was quite higher than the 1.3 % *E. coli* species reported from the Polish Notes and Coins (Kalita et al. 2013*)* indicating the difference in hygienic handling condition between African and European country. Although the magnitudes were differ, microbial contamination of currency were reported globally (Kalita et al. 2013; Awel et al. 2010; Barolia et al. 2011; Girma et al. 2014). The absence of *E. coli* in new currency used for control but its presence on those circulating once indicates, the currency under usage harboring *E. coli* and other microbial. Similarly, Kalita et al. (2013) also indicated presence of opportunistic microbial on the used Polish Notes and Coins but not on the new ones. About greater than 70 % of ETB were harboring *E. coli* and showed high and equal contamination within and among studies variables (location, food types, sources, ages and sex of handlers) of the currency in the studied district. However, the low *E. coli* rate on currency from open market (47.1 %) than from dairy station (87.2 %) at milk sellers (handlers) from Haramaya University and the surrounding indicated high prevalence of *E. coli* in dairy farm that can act as source of currency contamination through the milk. In Ethiopia, poor-currency-handling culture is widespread, and there is indiscriminate abuse of currency notes reported by Girma et al. (2014). Observation of *E. coli* on currency handled by vegetable handlers in the present study could also be contamination of the specific food from already contaminated dust, soil and water as well as from microflora of the body of handlers. Awel et al. (2010) and Barolia et al. (2011) also provided the same suggestion by tracing contaminant *E. coli* to dust, soil, water and the microflora of the body of handlers (hand, skin and others). Moreover, paper currency can acquire *E. coli* with droplets during coughing, sneezing, touching with contaminated hands or materials and from dirty surfaces at any point of placement. These was also shown by Ahmed et al. (2010) and Barolia et al. (2011). The Food and Agriculural Organization (2011) also suggested the persistence and growth ability of generic *E. coli* in many on foods. Thus, currency used for food marketing can acquire microbial including *E. coli* from already contaminated food items. Poor storage and handling, and frequent manual count activities also enhance currency cross contamination. Lamichane et al. (2009) and Sanjogita and Geeta (2014) provided similar suggestions.

With regards to specific ETB notes, *E. coli* harboring rate ranged from 60 to 85 % showing high and similar exposure of most currency denominations. The relatively lower prevalence on Birr 100 and Birr 50 than other denomination (the Birr 1, Birr 5 and Birr 10) are similar with previously reported from Jimma (13.4 % *Enterobacteriaceae*) by Girma et al. (2014) and from Axsum by Saripalli et al. (2014), both in Ethiopia. This could be due to frequent uses and high accesses of the latter notes which increase the risk of exposure for contamination. Diseases can spread through contact with fomites such as currency notes (Awel et al. 2010; El-Dars and Hassan 2005; Feglo and Nkansah 2010; Michaels 2002; Pope et al. 2002).

High susceptibility (97.7–100 %) of the tested isolates to chloramphenicol, oxytetracycline, trimethoprim-sulfamethoxazole and polymyxin-B indicated the efficacy of these drugs for the treatments of *E. coli* infection. High and similar susceptibility of *E. coli* to most of these drugs were also reported by Hiko et al. (2008) and Kibret and Abera (2011) in Ethiopia. However, the significantly high (83.7 %) resistance to neomycin could be due to frequent use the drug in medical sectors leading to development of resistant strain that contaminate the currency from such personnel.

## Conclusion

It was concluded that, the food which had already contaminated can be further act as source of contamination of currency used for exchange purposes among various food handlers. Again, contaminated currency can act as serious source for likelihood of harmful microbial including pathogenic *E. coli* strains. Theses may leads to cross-transmission of ready-to-eat food items such as fruits, bread and the related with possibility of consumer infection. Thus, regular disinfection, use of banks, post offices and ATM with local languages, as well as application of ultraviolet light and fumigation, with parallel replacement of damaged and worn-out notes and education of food handlers were recommended to reduce contamination. The study also provides information on the likely choice of therapeutic antimicrobial agent for infections that might arise from this organism in the area. Further study on the prevalence of other microbial on currency including on coins at other part of the country were also recommended.
